# Why Do You Dance? Development of the Dance Motivation Inventory (DMI)

**DOI:** 10.1371/journal.pone.0122866

**Published:** 2015-03-24

**Authors:** Aniko Maraz, Orsolya Király, Róbert Urbán, Mark D. Griffiths, Zsolt Demetrovics

**Affiliations:** 1 Department of Clinical Psychology and Addiction, Eötvös Loránd University, Budapest, Hungary; 2 Doctoral School of Psychology, Eötvös Loránd University, Budapest, Hungary; 3 Department of Personality and Health Psychology, Eötvös Loránd University, Budapest, Hungary; 4 Nottingham Trent University, Nottingham, United Kingdom; London School of Hygiene and Tropical Medicine, UNITED KINGDOM

## Abstract

Dancing is a popular form of physical exercise and studies have show that dancing can decrease anxiety, increase self-esteem, and improve psychological wellbeing. The aim of the current study was to explore the motivational basis of recreational social dancing and develop a new psychometric instrument to assess dancing motivation. The sample comprised 447 salsa and/or ballroom dancers (68% female; mean age 32.8 years) who completed an online survey. Eight motivational factors were identified via exploratory factor analysis and comprise a new Dance Motivation Inventory: Fitness, Mood Enhancement, Intimacy, Socialising, Trance, Mastery, Self-confidence and Escapism. Mood Enhancement was the strongest motivational factor for both males and females, although motives differed according to gender. Dancing intensity was predicted by three motivational factors: Mood Enhancement, Socialising, and Escapism. The eight dimensions identified cover possible motives for social recreational dancing, and the DMI proved to be a suitable measurement tool to assess these motives. The explored motives such as Mood Enhancement, Socialising and Escapism appear to be similar to those identified in other forms of behaviour such as drinking alcohol, exercise, gambling, and gaming.

## Introduction

Health experts constantly face the challenge of how to increase physical fitness and psychological wellbeing. Dancing can provide a strenuous but enjoyable way of exercising that can improve people’s level of fitness and to encourage a more active lifestyle. Dance is an activity that promotes fitness and improves aerobic and physical working capacity [1, 2]. Furthermore, there is much evidence to support the benefits of dancing including improvements in psychological wellbeing [3, 4], increased self-esteem [5], and anxiety reduction [6]. According to a recent study conducted on a nationally representative sample of the United States dancing is a common activity among adolescents, with a past-month prevalence rate of 20.9% [7]. However, we know very little about why people continue or discontinue to dance, or why dancing is chosen as a recreational sporting activity.

Exercise is ‘a sub-category of physical activity, that is planned structured purposeful and repetitive and has as a final or an intermediate objective which is the improvement or maintenance of physical fitness’ (p. 126.) [8]. Although dance is clearly a form of exercise [9, 10], it differs in a number of aspects. For example, dancing is closely linked to music and mostly requires the presence and physical closeness of a partner as opposed to most other exercise activities.

Recent research shows that motivation plays a substantial role in our leisure behaviour. For example, in the case of drinking alcohol, motives such as social, enhancement and coping explain up to 50% of the variance in adolescent alcohol use [11]. Motivation also plays an important (if not determining) role in the case of smoking cigarettes [12, 13] and in the use of ingesting other psychoactive substances [14, 15] as well as in gambling [16], online gaming [17] and exercising [18]. On the basis of studies examining these other leisure activities, the examination of the motivational background of dancing could be arguably just as important.

There have been very few empirical studies that have explored the motivations of dancing. Most studies have used a descriptive-qualitative method of assessment [19–22]. There is only one study that developed and tested a self-report questionnaire of dance motivation. Nieminen [23] created 25 items from dancers’ self-reports (N = 308) that loaded on four factors. The single inclusion criterion was a minimum of three years’ dance experience, although the mean number of years’ experience was nine years (and therefore the study mainly captured experienced dancers). The sample was largely heterogeneous and included many dance types (folk, ballet, ballroom-competitive, and modern). However, this approach is difficult to generalise to other types of dancers given that some of the items created are not applicable to recreational dancers (i.e., “preparing for a career”) while others are specific to certain genres (i.e. “travelling” as a motivation) and not to others. Furthermore, substantial cross-loadings in principal component analysis limit the usability of the separate scales. To the authors’ knowledge, a suitable instrument to assess the motivation of recreational social dancers has yet to be developed.

In addition, the majority of studies published on dance motivation have only examined professionals’ motivation to dance rather than recreational (social) dance motivation [19, 22]. However, motivation may be very different in recreational compared to professional dancers given that there are various self-selective processes on route to becoming a professional dancer [24]. Moreover, there is much evidence that recreational and professional athletes have very distinct motivations [25, 26]. For example, professional athletes are generally less motivated by mood enhancement and intrinsic factors (such as exercising for pleasure and satisfaction) that are important predictors of regular exercising among recreational athletes [27–29]. This is especially important because psychological factors mostly influence intrinsically motivated behaviour [30, 31] creating a possible point of intervention to enhance the drive to exercise or dance.

The aim of the present research study was two-fold. Firstly, the study aimed to uncover the underlying motivational components of social-recreational dancers. Secondly, the study aimed to operationalize the underlying dimensions found, and develop a scale to assess the identified dimensions. Additionally, the study explored the differences of motivation across gender and the level of dance activity. The study was also designed to improve upon the methodological shortcomings of earlier studies by using a large sample of dancers and control for possible mediating variables such as intensity and experience in the motives for dancing.

## Method

### Participants and procedure

The study aimed to capture individuals who participated in Latin dances (i.e., salsa, Latin or ballroom) for recreational and social purposes at least once a week. Data collection was carried out online. A link to the questionnaire was posted on the most popular Hungarian Latin dance website (latinfo.hu) and shared on *Facebook* between June and August 2013. A total of 688 began the survey of which 457 were completed. A further 10 were excluded because the respondents indicated they had never danced in the listed genres (i.e., salsa, Latin or ballroom) before. This resulted in 447 completed responses. Participants could only begin the questionnaire after providing informed consent to participate in the study. Identifying data were not collected to ensure anonymity. The study protocol was approved by the Institutional Review Board (IRB) of the Eötvös Loránd University.

### Measures

#### Dance Motivation Inventory (DMI)

The development of the 51-item list of dance motives was carried out over a number of stages. First, following a systematic literature review, two independent experts collected all statements that referred to the motivational basis of sport dance or exercise. This first stage identified 20 statements. At the same time, 11 dancers of varying experience were asked to list as many reasons and motives for dancing as possible. They were asked to complete the following sentence: “I dance because…” Overall, 74 motives were collected from these 11 individuals. In the next stage, the two lists of motives were merged, and duplicates and ambiguous items were removed. Any disagreement between the two experts was resolved by a third expert. Following this stage, a list of 51 items of possible motives for dance remained. Items of the DMI were evaluated by the study participants on a five-point scale (1 = I strongly disagree; 5 = I strongly agree).

#### Dance experience and intensity

Dance experience (or persistence) [20] was defined as the number of years that the participant had been actively involved in dancing, while intensity was operationalized as the number of hours spent in training and/or in a formal dance event in an average week.

#### Statistical Analysis

Statistical analysis comprised an exploratory factor analysis (EFA) with robust maximum-likelihood estimation (MLR) in MPlus 6.12 [32]. The goodness of fit was assessed by the root-mean-square error of approximation (RMSEA) and its 90% confidence interval (CI), and *p* value larger than 0.05 for test of close fit (Cfit>.05). Non-significant probability (Cfit) values are viewed as indicators of good model fit [33]. Additionally the χ^2^ test and its *p* value, and the comparative fit index (CFI) were evaluated. The χ^2^ test should be non-significant (*p* >. 05) for a close fit. However, this index is almost always significant in the case of large sample sizes. Therefore CFI as an alternative index of fit was also considered. Values greater than. 90 indicate an acceptable fit [34]. For the further development of the scale, those items were kept that loaded ≥.50 on only one factor, and loaded <.30 on any other factor.

The remaining statistical analyses were carried out with SPSS17 for Windows. The summary of items divided by the number of items the participant answered comprised the factors as scales. Pearson product-moment correlations were applied to assess associations between factors, and independent sample t-tests were used to assess differences between males and females. Linear regression analysis was used to identify the best motivational predictors of dance experience and intensity outcomes. Differences between motivational factors were assessed using paired t-tests. In order to perform a linear regression, multicollinearity was verified. As a rule of thumb, a VIF value greater than 4 would indicate inflated standard errors of regression coefficients [35]. In the current sample, none of the VIF values exceeded this limit: VIF values ranged between 1.40 (Escapism) and 2.01 (Self-confidence).

## Results

### Sample description

Approximately two-thirds of the sample (68%; n = 305) was female. The mean age was 32.8 years (SD = 8.6). The majority of the sample (70%) had graduate education, 28% had secondary school education, and the remainder (2%) reported having an education lower than secondary school. Just over one-quarter of the sample (29%) was still studying at an educational establishment. Just under three-quarters of the sample (72%) worked full-time, 15% were unemployed, and the remainder (13%) worked part-time or less. Just over one-third of the sample (38%) was single, 25% were in a relationship, 23% were married or co-habiting, 9% were in a more complicated relationship, and the remainder (5%) were divorced. In relation to dance experience, 9% of the participants had danced for 12 months or less, 26% for 1–2 years, 39% for 3–6 years, and 26% had danced for more than 6 years.

### Exploratory Factor Analyses

An exploratory factor analysis was performed with maximum-likelihood estimation and an oblique (Geomin) rotation to evaluate the factor structure of the 51 items on the sample (N = 447). A total of 6- to10-factor solutions were examined. RMSEA values were 0.066 [0.063–0.069] Cfit <. 0001 for the six-factor solution; 0.059 [0.056–0.062] Cfit <. 001 for the seven-factor solution; 0.055 [0.052–0.058], Cfit = 0.005 for the eight-factor solution; 0.050 [0.047–0.053] Cfit = 0.475 for nine factors, and finally 0.048 [0.044–0.051] Cfit = 0.871 for ten factors. Therefore, the nine-factor solution provided the first adequate (non-significant) Cfit value. Additional model fit indices for the nine-factor solution were also acceptable: χ^2^ = 1807.8 df = 852, *p*<.001; CFI = 0.915. Of the original 51 items, 29 met the aforementioned criteria for item selection (see Table 1). In the end, Factor 9 included only one item (Item 14), therefore this factor was excluded from further analyses.

**Table 1:** Factor structure of the Dance Motivation Inventory.

I dance…		Factor 1 *Fitness*	Factor 2 *Mood Enhancement*	Factor 3 *Intimacy*	Factor 4 *Socialising*	Factor 5 *Trance*	Factor 6 *Mastery*	Factor 7 *Self-confidence*	Factor 8 *Escapism*	Factor 9
10.	… to watch my lines	**.98**	-.03	.05	-.01	.02	-.07	.00	-.01	.00
11.	… to be healthy	**.80**	.02	.02	.03	-.02	.06	.06	-.01	.00
3.	… to be fit	**.79**	.06	-.03	.01	-.07	.06	.04	-.05	.18
14.	… to exercise	**.65**	.00	-.02	-.01	.08	.16	.03	.11	.02
22.	… because I enjoy it	-.03	**.79**	-.01	.02	.03	-.03	-.01	-.05	.11
25.	… because dancing improves my mood	-.02	**.77**	.06	-.03	-.05	.02	.05	.20	.02
17.	… because it fills me up with energy	.10	**.61**	-.08	.02	.25	.00	.09	-.01	-.03
27.	… because girls are pretty / boys are handsome	-.01	-.03	**.79**	.05	.03	.09	-.13	-.08	.05
7.	… because I am looking for a relationship	.00	-.09	**.71**	.07	-.09	.18	.00	.05	.01
9.	… because I like being physically close to another human being	.01	.02	**.62**	-.07	.22	.01	.13	-.16	.06
19.	… because I am looking for a sex partner	.00	-.26	**.57**	-.02	.04	-.03	-.08	.04	.00
30.	… because it makes easy to socialise	.01	-.01	**.57**	.22	-.12	.10	.12	.14	-.14
6.	… because I like the company	.00	.18	-.01	**.77**	.01	.02	-.07	.02	.04
5.	… because I am surrounded by people who think like me	.02	-.09	-.05	**.77**	.08	.01	.06	-.05	.14
46.	… because I can meet many people like me	-.02	-.03	.05	**.72**	.00	.04	.05	.09	.09
13.	… because I can experience a trance-like state	.01	-.03	.05	.02	**.83**	.00	.07	.01	-.03
16.	… because I can experience ecstasy	.02	-.03	.10	.09	**.83**	-.04	.02	-.02	.02
18.	… because it feels like floating	.06	.24	-.04	.05	**.64**	.00	.04	.04	-.02
51.	… because I can experience an altered state of mind	-.03	.06	-.01	-.05	**.78**	.12	-.07	.18	-.01
29.	… because it improves my coordination	.13	.01	.02	.08	-.02	**.61**	.10	.01	.01
21.	… because I like being in control of my body	-.04	-.02	.01	-.02	.24	**.57**	.15	-.04	.04
33.	… because I constantly improve	.04	.29	-.04	.09	.00	**.50**	-.01	-.01	.22
15.	… because dancing brings out the man/woman within me	.03	.16	.02	-.04	.12	.06	**.68**	-.06	.05
44.	… because I feel sexy when I dance	.08	.04	.12	-.12	.07	-.05	**.55**	.13	.21
12.	… because dancing improves my self-esteem	.16	.06	-.04	.12	-.02	-.03	**.53**	.13	.20
48.	… to avoid feeling the blues	.05	.11	-.01	-.03	-.01	.02	-.09	**.76**	.10
47.	… because otherwise my life would be empty	-.08	.02	.03	.05	.07	.01	-.11	**.69**	.14
45.	… because when I dance, I don't have to deal with my everyday problems	.08	.20	.00	-.03	.00	-.01	.19	**.59**	-.11
41.	… because I feel that I would miss something if I didn't dance	-.01	-.08	-.06	.09	.04	.05	.16	**.51**	.01
	Factor determinacies	0.97	0.95	0.93	0.94	0.96	0.87	0.91	0.93	
	Cronbach’s alpha	0.91	0.81	0.80	0.85	0.90	0.73	0.81	0.79	
	Mean	2.47	3.57	1.58	2.71	2.39	2.75	2.94	2.14	
	SD	0.94	0.64	0.91	1.02	1.23	0.94	0.94	1.06	
**Excluded:**										
1.	… because it gives me a feeling of success	.05	.03	.01	.16	.01	.05	.19	.00	**.52**
2.	… because when I dance, I feel happy	.03	.64	-.04	.01	.10	.05	.05	.03	.32
4.	… because I receive many positive feedback	.08	.06	.10	.13	.00	.12	.11	-.03	.46
8.	… because dancing gives me pleasure	.04	.19	.41	.05	.29	.03	.00	.03	.16
20.	… because I constantly expand my physical limits	.06	-.08	-.03	-.02	.37	.53	.03	.06	.08
23.	… because I get to know new people	.04	.07	.33	.50	-.08	.06	.01	.00	-.08
24.	… because I can meet my old friends/acquaintances	.04	.11	.09	.49	.02	.04	-.02	.01	-.09
26.	… because I can communicate with my partner beyond words	-.07	.05	.22	.12	.21	.10	.41	-.07	-.08
28.	… because I like the predictable moves	.01	-.06	.20	.09	.01	.42	.05	.08	-.01
31.	… because I enjoy watching others dance	.00	.26	.16	.16	.02	.41	-.11	.05	-.13
32.	… because dancing reduces daily stress	.05	.50	-.02	-.01	.04	.18	.03	.34	-.15
34.	… to enrich my everydays	.05	.37	.10	.13	-.01	.08	.08	.26	.06
35.	… to show off my dancing skills to others	.01	-.18	.11	.03	.09	.06	.15	.15	.48
36.	… to express myself	-.03	.07	-.06	.04	.34	.08	.33	.02	.20
37.	… because I like the atmosphere of the parties	.01	.18	.19	.35	.22	-.06	-.02	.01	.04
38.	… because when I dance, I don't feel lonely	-.08	.00	.25	.02	.08	-.10	.15	.49	.03
39.	… to lose weight	.51	-.30	-.01	.02	.08	-.03	-.01	.34	-.04
40.	… because it reduces my shyness	-.01	.00	.08	.04	-.02	.02	.44	.46	-.10
42.	… because the self-confidence I gain during dancing has a good effect on other areas in my life	.00	-.03	-.08	.10	.02	.01	.54	.32	.11
43.	… because I like leading my partner / I like to be led	.00	-.01	.27	-.05	.14	.06	.29	.07	.01
49.	… because others respect me when I tell them that I dance	.01	-.21	.06	-.05	-.06	.24	.04	.40	.30
50.	… because my dancing constantly improves	-.02	.06	.01	.01	-.01	.40	-.08	.26	.34

Note: Exploratory factor analysis was conducted with maximum likelihood estimation, oblique rotation. Factor loadings are in bold

### Labelling of factors

Four items belonged to the first factor (*Fitness*) as they referred to dancing in order to keep fit and healthy. The second factor (*Mood Enhancement*) contained three items and referred to the mood improving and energising nature of dancing. The five items belonging to the third factor (*Intimacy*) referred to the attractiveness of outfits, searching for relationships and sexual partners, and physical closeness to another person. The fourth factor (*Socialising*) referred to items relating to being in good company and being with like-minded people. The fifth factor (*Trance*) referred to experiences of trance, ecstasy, floating, and dancing as a way to reach altered state of mind. The sixth factor (*Mastery*) included motivations that arose from the improvement of coordination, and body movements, as well as increasing control of one’s own body. The seventh factor (*Self-confidence*), contained three items referring to the feeling of sexiness and improved self-esteem. The final factor (*Escapism*) contained four items that referred to the avoidance of emptiness, bad mood, and everyday problems. All factors have acceptable internal consistencies (see Table 1).

### Motivational factors: Differences and correlation

Mood Enhancement scores were significantly higher than the other factor scores (ranging from t_intimacy_ = -36.57 to t_self-confidence_ = -15.93 all *p*<0.05). Results indicated there was overlap between most factors (Table 2). The highest correlation was between Self-confidence and Trance (r = .56, *p*<.01), Self-confidence and Mastery (r = .53, *p*<.01), and Self-confidence and Mood Enhancement (r = .51, *p*<.01).

**Table 2:** Factor correlation matrix.

	1	2	3	4	5	6	7	8
**Fitness (1)**	1.00							
**Enhancement (2)**	.34**	1.00						
**Intimacy (3)**	.06	-.07	1.00					
**Socialising (4)**	.35**	.31**	.31**	1.00				
**Trance (5)**	.26**	.46**	.19**	.28**	1.00			
**Mastery (6)**	.47**	.38**	.22**	.49**	.39**	1.00		
**Self-confidence (7)**	.46**	.51**	.22**	.38**	.56**	.53**	1.00	
**Coping (8)**	.29**	.33**	.23**	.35**	.38**	.41**	.44**	1.00

*Note*:

**p<.*01*

### Gender differences

**Fig 1:**
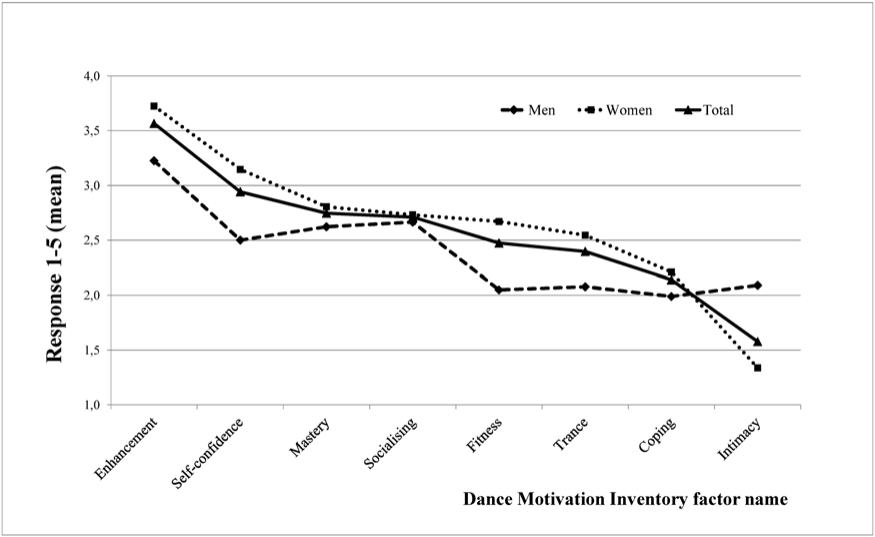
Gender differences in dance motivation.

As shown in Fig 1, the strongest motivational factor was Mood Enhancement, followed by Self-confidence. Women were more likely to dance for reasons of Fitness (t = -5.81 *p*<.001), Mood Enhancement (t = -8.22 *p*<.001), Trance (t = -3.80 *p*<.01), Self-Confidence (t = -7.10 *p*<.001) and Escapism (t = -2.05 *p*<.05) than men. Men on the other hand were mostly motivated by Intimacy (t = 8.82 *p*<.001). There was no significant difference between males and females regarding Socialising (t = -0.648 *p* = .518) and Mastery (t = -1.92 *p* = .055).

### Dance activity and motivation

In the next step, all factors were entered in a linear regression model with the indicators of dance activity (i.e., Experience and Intensity) as dependent variables. Experience was not significantly predicted by any of the DMI factors (F = 2.23 *p* = .28, R^2^ = 0.004). On the other hand, Intensity was predicted by three of the motives (F = 6.76 *p*<.001, R^2^ = 0.11, adjusted R^2^ = 0.09): Intimacy (*ß* = 0.17, p<.001), Socialising (*ß* = 0.15, *p*<.01), and Mastery (*ß* = 0.18, p<.01).

## Discussion

The aim of the present study was to gain deeper knowledge of and to operationalize the motivational basis of recreational social dancing. Consequently, the Dance Motivation Inventory (DMI) was developed and proved to be a reliable tool to assess the motivation to dance. The DMI contains 29 items that loaded on eight factors: Fitness, Mood Enhancement, Intimacy, Socialising, Trance, Mastery, Self-confidence and Escapism. Being aware of the different motivational factors of dance will help increase participation in dance to enable individuals profit from the health benefits of the activity.

Compared to the only other previously published dance motivation inventory tested on experienced dancers [23], Fitness and Achievement (Mastery) was replicated, but Self-expression did not emerge in the current sample. Furthermore, Social contact separated into two distinct factors, Socialising and Intimacy. In addition, four additional factors were identified (i.e., Mood Enhancement, Self-confidence, Trance and Escapism) compared to previous results. These factors appear to be specific to recreational social dancers’ motivation as opposed to those of experienced dancers’. Escapism is a particularly important motivational factor given that it is linked to problematic, even addictive behaviour especially when Mood Enhancement as a motivation is also present [36].

Interestingly, many of the previously described motivational factors related to other behaviours (e.g., exercise, gambling, gaming or drinking alcohol) also appeared among the eight dimensions identified in this study. Three factors, Mood Enhancement, Socialising, and Escapism are present in dance motivation as well as across many other behaviours such as exercise [37], drinking alcohol [38], online gaming [17, 39], gambling [40, 41] and cannabis use [42]. These findings provide support that there are similar motivational factors behind various activities that also play an important role in dancing. Furthermore, Mastery emerges similarly to Skill development in online gaming [17]. Trance resembles Fantasy identified in online gaming [17] and Expansion in cannabis use [42]. However, two factors–Self-confidence and Intimacy–appear to be specific to dancing. The motive of Intimacy differentiates DMI from other sport-related inventories such as the Exercise Motivations Inventory [37]. It therefore appears that the physical closeness of dance partners is a strong determinant of dance motivation compared to other forms of exercise.

Mood Enhancement was by far the strongest motivational factor for dance activity similar to exercise [43]. Dancing is a recreational activity which is pursued dominantly to improve one’s mood and reflects the stress-reducing capability of the activity [29]. Programs that have the aim of increasing participation in dancing should therefore focus on the mood-enhancing and self-confidence improving nature of dancing. On the other hand, Mastery in exercise [44] and Escapism in gaming [45] are known to be risk factors for problematic behaviour (dependence), and therefore the motivational background of dance addiction [46] could also be a future topic of research.

The level of dance activity was only partially linked to motives. Experience did not appear to be related to motivation, which is contrary to the authors’ expectations [24–29]. Perhaps accounting for the nature of experience (active years vs. duration from first experience) would further clarify the relationship between dance experience and motivation. On the other hand, Intensity (i.e., the number of weekly practices) was predicted by the motives for Intimacy, Socialising, and Mastery. The opportunity for social and physical contact appears to be just as important as improving one’s skills when it comes to the frequency of dancing.

The present study has both strengths and limitations. Strengths include the large and homogenous sample of social recreational dancers. On the other hand, findings obtained via a homogenous sample limits generalizability of results to other genres of dance. Another limitation concerns the self-selected and self-reported nature of the data. Results concerning the motivational background of dancing require confirmation among different independent samples. Future studies should also address the question of causality between motivational factors and intensity, given that cross-sectional data is unsuitable to establish causality.

Dancing is a popular form of physical exercise and studies (outlined earlier in the paper) clearly show that dancing can decrease anxiety, increase self-esteem, and improve psychological wellbeing. Overall, the most important aspect of the present study is that, on the basis of the explored motivational background of recreational social dancers, a research instrument has been developed that can serve as a reliable tool for stimulating future research. Additional studies are needed to describe and compare different types of dancing along with their motivational basis. Another objective of future research in this field should be to define the relationship between specific motivational dimensions and different personality traits or characteristics.

## Supporting Information

S1 Appendix The Dance Motivation Inventory. Instructions: There are a number of reasons why people choose to dance. Some reasons are listed below. Why do you dance? Please answer from 1 to 5 where 1 = I strongly disagree, 2 = I disagree, 3 = I neither agree nor disagree, 4 = I agree, 5 = I strongly agree. There is no right or wrong answer. We are only interested in your motives for dancing. Key: *Fitness*: 12, 20, 21 and 9; *Mood Enhancement*: 22, 27 and 2; *Intimacy*: 13, 29, 18, 6 and 25; *Socialising*: 4, 14 and 15; *Trance*: 28, 10, 19 and 5; *Mastery*: 23, 1 and 7; *Self-confidence*: 16, 8 and 25; *Escapism*: 3, 17, 14 and 26.
(DOCX)
