# Supplementary material for: Why Do You Dance? Development of the Dance Motivation Inventory (DMI)
Source: PLoS One. 2015 Mar 24;10(3):e0122866. doi: 10.1371/journal.pone.0122866 (PMC4372397; doi:10.1371/journal.pone.0122866)
Supplement: S1 Appendix — Instructions: There are a number of reasons why people choose to dance. Some reasons are listed below. Why do you dance? Please answer from 1 to 5 where 1 = I strongly disagree, 2 = I disagree, 3 = I neither agree nor disagree, 4 = I agree, 5 = I strongly agree. There is no right or wrong answer. We are only interested in your motives for dancing. Key: Fitness: 12, 20, 21 and 9; Mood Enhancement: 22, 27 and 2; Intimacy: 13, 29, 18, 6 and 25; Socialising: 4, 14 and 15; Trance: 28, 10, 19 and 5; Mastery: 23, 1 and 7; Self-confidence: 16, 8 and 25; Escapism: 3, 17, 14 and 26. (DOCX) [file pone.0122866.s001.docx]

**Supplementary Appendix 1.: The Dance Motivation Inventory**

I dance…

1. … because I like being in control of my body

2. … because it fills me up with energy

3. … to avoid feeling the blues

4. … because I like the company

5. … because I can experience an altered state of mind

6. … because I am looking for a sex partner

7. … because I constantly improve

8. … because I feel sexy when I dance

9. … to exercise

10. … because I can experience ecstasy

12. … to watch my lines

13. … because girls are pretty / boys are handsome

14. … because I am surrounded by people who think like me

14. … because when I dance, I don't have to deal with my everyday problems

15. … because I can meet many people like me

16. … because dancing brings out the man/woman within me

17. … because otherwise my life would be empty

18. … because I like being physically close to another human being

19. … because it feels like floating

20. … to be healthy

21. … to be fit

22. … because I enjoy it

23. … because it improves my coordination

24. … because it makes easy to socialise

25. … because dancing improves my self-esteem

26. … because I feel that I would miss something if I didn't dance

27. … because dancing improves my mood

28. … because I can experience a trance-like state

29. … because I am looking for a relationship

Instructions: There are a number of reasons why people choose to dance. Some reasons are listed below. Why do you dance? Please answer from 1 to 5 where 1=I strongly disagree, 2=I disagree, 3=I neither agree nor disagree, 4=I agree, 5=I strongly agree. There is no right or wrong answer. We are only interested in your motives for dancing.

Key: *Fitness*: 12, 20, 21 and 9; *Mood Enhancement*: 22, 27 and 2; *Intimacy*: 13, 29, 18, 6 and 25; *Socialising*: 4, 14 and 15; *Trance*: 28, 10, 19 and 5; *Mastery*: 23, 1 and 7; *Self-confidence*: 16, 8 and 25; *Escapism*: 3, 17, 14 and 26
